# Deciphering the Pharmacological Properties of Methanol Extract of *Psychotria calocarpa* Leaves by In Vivo, In Vitro and In Silico Approaches

**DOI:** 10.3390/ph13080183

**Published:** 2020-08-06

**Authors:** Tahmina Akter Bristy, Niloy Barua, Abu Montakim Tareq, Shahenur Alam Sakib, Saida Tasnim Etu, Kamrul Hasan Chowdhury, Mifta Ahmed Jyoti, Md. Arfin Ibn Aziz, A.S.M. Ali Reza, Elisabetta Caiazzo, Barbara Romano, Syed Mohammed Tareq, Talha Bin Emran, Raffaele Capasso

**Affiliations:** 1 Department of Pharmacy, International Islamic University Chittagong, Kumira, Chittagong 4318, Bangladesh; tahminabristy4@gmail.com (T.A.B.); niloybaruaniloy@gmail.com (N.B.); montakim0.abu@gmail.com (A.M.T.); saidatasnim96@gmail.com (S.T.E.); kamrulhasan73132@gmail.com (K.H.C.); mifta_ahmed@yahoo.com (M.A.J.); arfinibnaziz151085@gmail.com (M.A.I.A.); alirezaru@gmail.com (A.S.M.A.R.); 2 Department of Theoretical and Computational Chemistry, University of Dhaka, Dhaka 1000, Bangladesh; sakibhasaniiuc@gmail.com; 3 Department of Pharmacy, School of Medicine, University of Naples Federico II via Domenico Montesano, 49 80131 Naples, Italy; elisabetta.caiazzo@unina.it (E.C.); barbara.romano@unina.it (B.R.); 4 Department of Pharmacy, BGC Trust University Bangladesh, Chittagong 4381, Bangladesh; 5 Department of Agricultural Sciences, University of Naples Federico II, 80055 Portici, Italy

**Keywords:** *Psychotria calocarpa*, Rubiaceae, methanol extract, antidepressant, antinociceptive, antidiarrheal, antioxidant, molecular docking

## Abstract

The present study explores the neuropharmacological, antinociceptive, antidiarrheal, antioxidant, thrombolytic and cytotoxic activity of methanol extract of *Psychotria calocarpa* leaves (MEPC). In anxiolytic activity testing of MEPC by elevated plus maze test, hole–board test and light–dark test, the extract exhibited a dose-dependent reduction of anxiety while the open field test observed a decreased locomotion. The administration of MEPC revealed a significant dose-dependent reduction of depressant behavior in forced swimming and tail suspension test. Additionally, the antinociceptive and antidiarrheal activity exposed a significant reduction of nociception and diarrheal behavior at the highest dose. In addition, a strong antioxidant activity was observed in DPPH-free radical-scavenging assay (IC_50_ = 461.05 μg/mL), total phenol content (118.31 ± 1.12 mg) and total flavonoid content (100.85 ± 0.97 mg). The significant clot–lysis activity was also observed with moderate toxicity (LC_50_ = 247.92 μg/mL) level in the lethality assay of brine shrimp. Moreover, in silico molecular docking study showed that the compound Psychotriasine could offer promising active site interactions for binding proteins. Furthermore, ADME/T and toxicological properties of the compound satisfied the Lipinski’s rule of five and Veber rules for drug-like potential and toxicity level. Overall, MEPC had a potential neuropharmacological, antinociceptive, antidiarrheal and antioxidant activity that warranted further investigation.

## 1. Introduction

Depressive disorders are common psychiatric disorders characterized by inflammatory conditions, oxidative stress, overproduction of nitric oxide and imbalance of antioxidant [1], whereas 121 million people affected by it [2]. According to the World Health Organization (WHO) and Years Lived with Disability (YLDs) states that depression is a major cause of disability and fourth leading psychiatric disorders in the world [2]. Depression may be probably comorbid with the other disorders for example obesity, anxiety, diabetes, Alzheimer’s disease, schizophrenia [3]. Oxidative stress considered for the major cause of depressive disorders. Hence, it is an important therapeutic target for the treatment of depression and its associated psychiatric disorders [3].

Oxidative stress (OS) is only the imbalance of oxidants and antioxidants, whereas it framed as a typical result of aerobic metabolism [4]. Both enzymatic (superoxide dismutase, catalase, glutathione peroxidase) and nonenzymatic (Vitamin C, Vitamin E, flavonoids) techniques are engaged with the antioxidant defense and antioxidant efficacy of any particle or molecule relies upon the cooxidant [4]. The oxidative stress is typically a complex process, which occurs due to the production of reactive nitrogen species (RNS) and reactive oxygen species (ROS) [5,6]. ROS is a continuous generation process of body metabolism, which helps in the management of different biologic and pathologic process, namely, immune system, lipid peroxidation, phagocytes activation [7,8]. Additionally, overproduction of ROS may cause oxidative damage by targeting the weak position of the components such as unsaturated fatty acids in membranes, thiol groups in proteins [9,10]. Several chronic health diseases are reported due to the overproduction of lipid peroxidation. According to several studies, free radical may cause several neuropsychiatric disorders and also mediate a neuronal dysfunction which led to schizophrenia with some other major depression [11,12,13]. Oxidative stress has a significant influence in the advancement of chronic and degenerative health diseases, for example, malignant growth [14], joint inflammation, aging, immune system issue, cardiovascular and neurodegenerative ailments [15]. The human body has a few mechanisms to neutralize oxidative stress by antioxidants, which are either provided through natural foods or additionally supplements [14,16]. Vitamin C and E together act as antioxidant defense, whereas the combinations of vitamin E/C (400 IU: 500 mg) are effective against the schizophrenia [17]. In addition, the N-acetyl-cysteine (NAC) twice per day (1 g) is useful for bipolar disorder [18] and even in depression [13]. Such investigations have likewise opened the potential roads of new treatment systems utilizing antioxidants as adjunctive treatment in the neuropsychiatric disorders and also naturally derived food substances have been used as a great potential over the last two decades and numerous biologic activities demonstrating promising aspect on anti-inflammatory, anti-apoptotic and modulatory effects [19].

In anxiety disorders, benzodiazepines are gradually substituted by antidepressants, which are not only useful in depression, but also in anxiety disorders for acute and long-term treatment [20]. Although, the tricyclic antidepressants and monoamine oxidase inhibitors are first generation antidepressant drugs, which poses toxicity in higher dose with several undesirable adverse effects and slow onset of action [21,22]. However, the new generation (second and third) antidepressant drugs (selective serotonin reuptake inhibitors and serotonin and norepinephrine reuptake inhibitors) are more targets selective. However, they also reported several major adverse effects, including gastrointestinal, CNS problems, seizure, insomnia, dry mouth and elevation of blood pressure [22]. So, researchers are trying to explore new potential lead compounds from medicinal plants having several biologic properties, whereas early tests (in vivo and in vitro) was followed to evaluate the possible activity and toxicity level of the lead compounds [21]. Three visual methodologies, including surface plasmon resonance, scintillation proximity assay and isothermal titration calorimetry are used to identify whether the compounds bind to the receptor. Later, the virtual screening, medicinal chemistry, combinatorial chemistry and computer-aided drug design (molecular docking, ADME/T) was followed to develop a new lead compound [21]. Despite this long and time-consuming process, natural products are the topic of interest in the treatment of several diseases. Hence, our present study design for biologic properties of *Psychotria calocarpa* using in vivo and in vitro assay with in silico study to explore a potential lead compound for chronic diseases.

*Psychotria calocarpa* belongs to the Rubiaceae family, which is locally known as ranga bhutta (Tanchangya, Chittagong, Bangladesh) [23]. Plants of the genus *Psychotria* have been used in folk medicine for the treatment of constipation in Malaysia [24]. The local Tanchangya community of Bangladesh used the plant in the treatment of hysteria. A compound named psychotriasine was isolated from the leaves of *P. calocarpa* by NMR analysis [25]. However, there is no scientific report on the biologic activity of *P. calocarpa* leaves. Hence, the present study has been designed to evaluate the biologic properties, namely, the anxiolytic, locomotor, antidepressant, antinociceptive, antidiarrheal, antioxidant, thrombolytic and cytotoxic activities as well as the semiqualitative phytochemical analysis of the methanol extract of *P. calocarpa* leaves. The objective of the study is to find a lead compound from *P. calocarpa* in moderating neuropsychiatric disorders by suppressing the oxidative stress signaling. As the plant was reported to contain one compound by NMR analysis, we used the computational studies using molecular docking, absorption, distribution, metabolism, excretion and toxicity (ADME/T) and toxicological properties analysis of the compound to understand the selected activities.

## 2. Results and Discussion

Plant species have demonstrated therapeutic promises for the treatment of neurological issue [26]. Phytochemicals are essential parts in protecting neural cells from inflammation and oxidative stress-related with aging, acute and chronic brain diseases [27]. In our present study, the semiqualitative phytochemical analysis of the methanol extract of *P. calocarpa* leaves was revealed several secondary metabolites, namely, the alkaloid, glycosides, tannins, saponins, resins, flavonoid and phenols (Table S1).

### 2.1. Effect of Methanol Extract of P. calocarpa on Anxiolytic Activity

**Figure 1:**
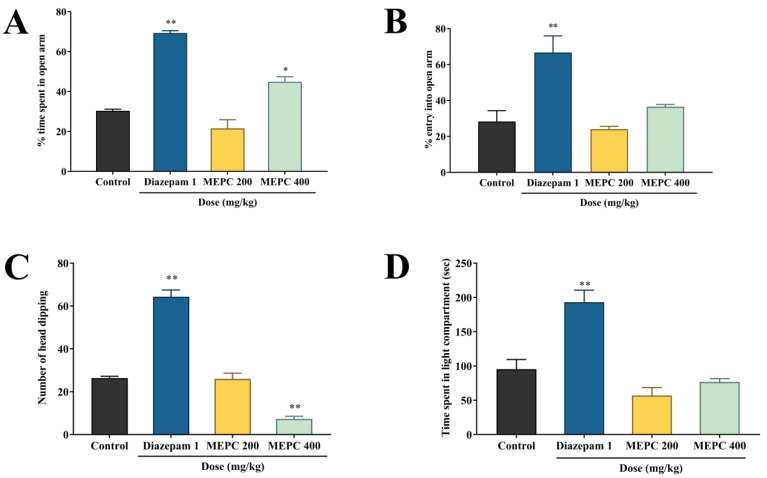
Anxiolytic activity of methanol extract of *P. calocarpa* (MEPC) and diazepam on elevated plus maze test, hole–board test and light and dark box test in mice. (**A**) % time spent in open arm, (**B**) % of entry into open arm, (**C**) number of head dipping and (**D**) time spent in light compartment. Values represented as mean ± SEM (*n* = 5). * *p* < 0.01 and ** *p* < 0.001 considered as significantly different from the control (Dunnett’s test).

For the preliminary anxiolytic activity analysis, the light–dark box (LDB), hole–board test (HBT) or elevated plus maze (EPM) tests were mostly used [28]. The increased level of GABAergic neurotransmission systems in the brain caused anxiolytic effects [29]. The EPM test is usually utilized for the assessment of anxiolytic substance in animals [30]. In EPM, a significant activity in dose-dependent manner was observed for time spent in the open arm, whereas the MEPC (400 mg/kg) exposed 44.89 ± 2.50% (*p* < 0.01) increased time spent in open arm compared with the control (30.33 ± 0.88%). For open arm entries, a statistically insignificant dose-dependent activity was observed by MEPC (200 and 400 mg/kg), whereas the standard drug diazepam exposed 66.67% ± 9.27% (*p* < 0.001) open arm entries. The effect of methanol extract of *P. calocarpa* on anxiolytic activity by EPM test was represented in Figure 1A,B. The HBT is the basic technique to assess the response of the animal to an unfamiliar condition and generally used to evaluate the emotionality and anxiety reactions in animals [31]. The higher number of head dipping in the hole of the apparatus indicates the effects of anti-anxiety [32]. MEPC at 200 mg/kg demonstrated a higher number of head dipping (26.0 ± 2.65) than at 400 mg/kg (7.33 ± 1.20). The standard drug diazepam revealed 64.33 ± 3.16 (*p* < 0.001) mean numbers of head dipping compared to the control (26.33 ± 0.88). The result was represented in Figure 1C. The LDT is likewise a favored model for assessing anxiolytic or anxiogenic drugs, where the animals revealed a strong tendency towards exploring the new condition. The lighted open areas of the apparatus have unresponsive properties that restrain this exploratory behavior of animals. At the point when given a decision between light or dark one, the animals will emphatically support the dark region. Anxiolytic substance used in this case to invert this inclination [31]. In addition, MEPC demonstrated a statistically insignificant anxiolytic activity in LDT, where 400 mg/kg showed highest time spent in light compartment (76.60 ± 4.82) than at 200 mg/kg (57.0 ± 11.63). The standard drug diazepam revealed a significant (*p* < 0.001) mean time spent in light compartment (193.0 ± 17.58) compared to the control (95.33 ± 14.17). The result was represented in Figure 1D.

From the above findings, the open arm time spent in EPM revealed a significant (*p* < 0.001) anxiolytic behavior at 400 mg/kg dose of MEPC, but another parameter (entries into open arm) showed a statistically insignificant activity. A similar result was observed for the HBT and LDT, whereas both doses of MEPC demonstrated anxiogenic-like effects. In contrast, both doses of MEPC findings were lower than the control group. These outcomes represent the anxiogenic nature of MEPC, which require further testing of these variables in the behavioral study.

### 2.2. Effect of Methanol Extract of P. calocarpa on Locomotor Activity

**Figure 2:**
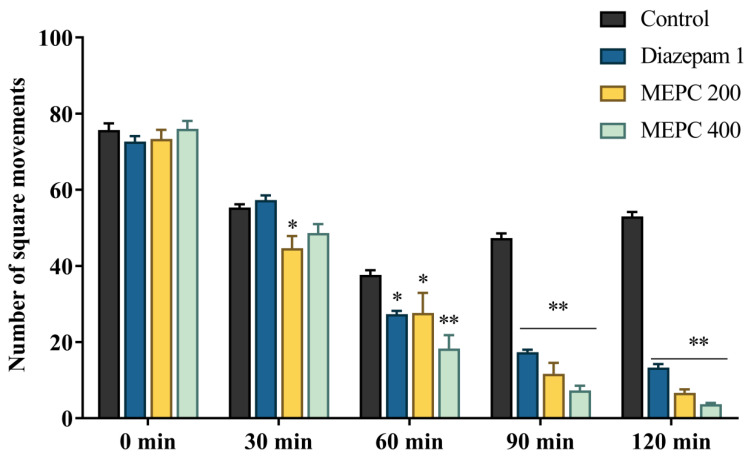
Locomotor activity of methanol extract of *P. calocarpa* (MEPC) and diazepam on open field test in mice. Values represented as mean ± SEM (*n* = 5). * *p* < 0.01 and ** *p* < 0.001 considered as significantly different from the control (two-way ANOVA with repeated measures).

The open field test (OFT) used to assess the locomotor and exploratory behavioral activity of mice/animals [33] and also useful in the evaluation of antidepressant like-behaviors [34]. Locomotion is considered as an index of alertness, whereas a decreased locomotion affects guide as central nervous system (CNS) depressant activity [35]. The locomotor activity of MEPC was evaluated by OFT, where a significant dose-dependent reduction in the movements of the square was observed by the 200 and 400 mg/kg in comparison with the control group. The reduction of the square movements was observed from the 30 min to 120 min, as shown in Figure 2. The decreased behavior of mice after administration of MEPC can be effective therapeutic substances, which may improve the motor function [36] and suppressed the active locomotion of the mice in OFT. MEPC was demonstrated a significant dose dependent reduction of locomotion which may probably show sedative and CNS depressant effects by stimulating the γ-aminobutyric acid-ergic (GABAergic) inhibition by hyperpolarization. These effects may lead to the reduction of neuronal firing or activation of γ-aminobutyric acid (GABA) receptors [37].

### 2.3. Effect of Methanol Extract of P. calocarpa on Antidepressant Activity

**Figure 3:**
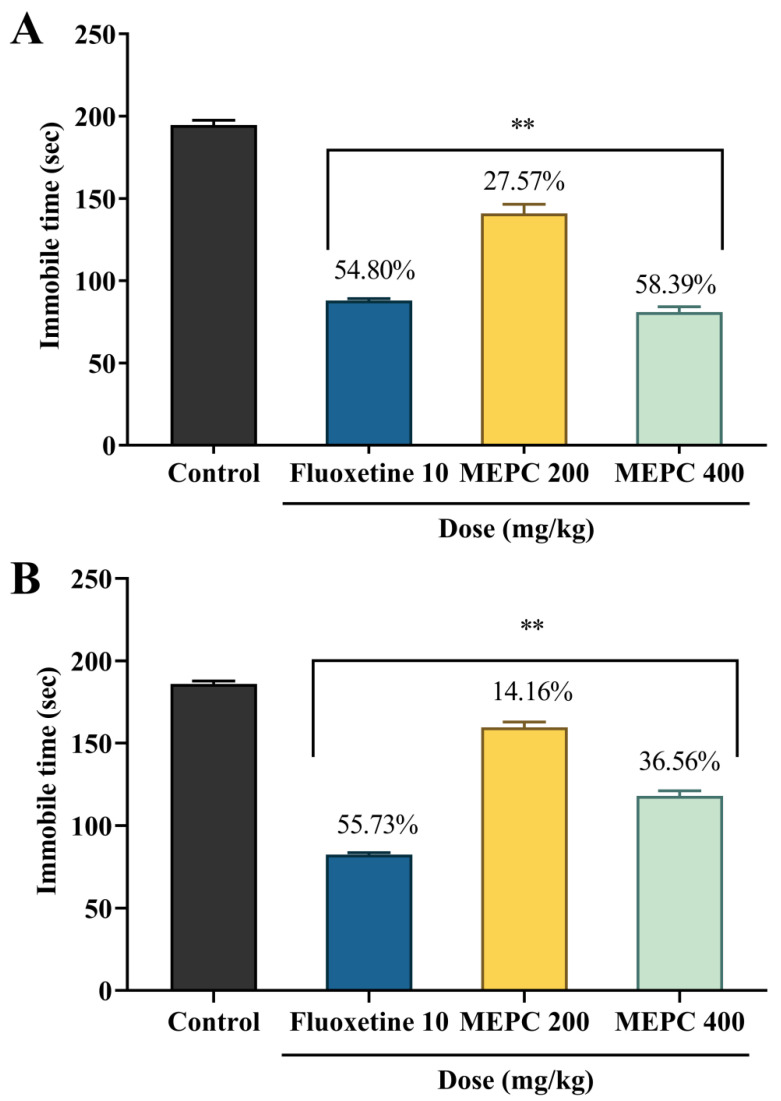
Antidepressant activity of methanol extract of *P. calocarpa* (MEPC) and fluoxetine on (**A**) forced swimming test and (**B**) tail suspension test. Values represented as mean ± SEM (*n* = 5). ** *p* < 0.001 considered as significantly different from the control (Dunnett’s test).

Anxiety and depression are connected and generally act as concurrent states and treatment of the two states positively influence the result of the therapy [38]. Serotonergic antidepressants or SSRIs are used as first-line treatment for patients with depression and also reported to have noteworthy anxiolytic impacts [39]. A few classes of medications that alter serotonin (5-HT) neurotransmission have recently been investigated for their effect in both depression and schizophrenia [40]. Several medicinal plants reported to have both anxiolytic and antidepressant effects, namely; *M. angolensis* [41], *N. sativa* [42] *L. angustifolia* [43]. Based on this, the antidepressant effect of methanol extract of *P. calocarpa* was assessed by forced swimming test (FST) and tail suspension test (TST). In statistical analysis, both FST and TST exhibited an extremely significant (*p* < 0.001) reduction of immobility time observed for MEPC (200 and 400 mg/kg) and standard drug fluoxetine against the control. In FST, the percentage of decrease in immobility for 400 mg/kg dose of MEPC was 58.39%, which was higher than the standard drug fluoxetine (54.80%). In TST, the percentage of decrease in immobility for 400 mg/kg dose of MEPC and the standard drug fluoxetine was revealed 36.56% and 55.73%, respectively. The result was represented in Figure 3.

### 2.4. Effect of Methanol Extract of P. calocarpa on Antinociceptive Activity

**Figure 4:**
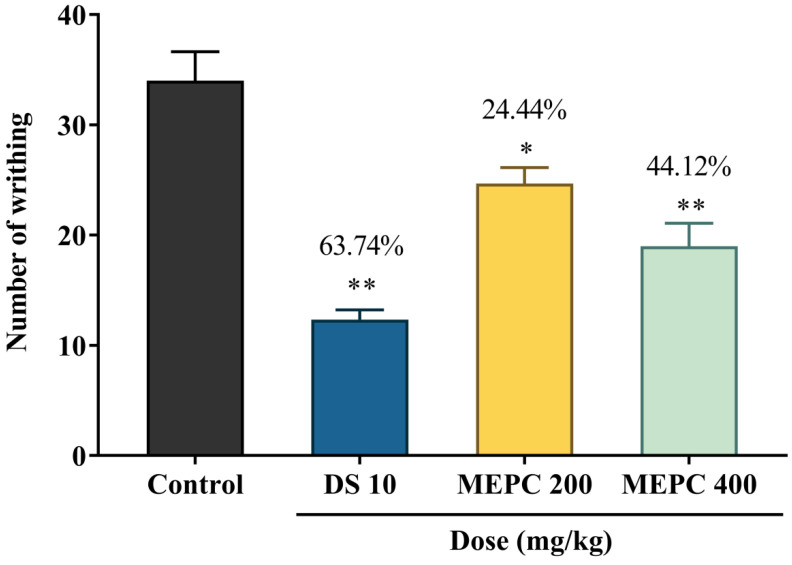
Antinociceptive activity of methanol extract of *P. calocarpa* (MEPC) and diclofenac sodium (DS) in acetic acid-induced writhing test in mice. Values represented as mean ± SEM (*n* = 5). * *p* < 0.01 and ** *p* < 0.001 were considered as significantly different from the control (Dunnett’s test).

The antinociceptive activity of MEPC was evaluated by the writhing response, where the pain in mice was induced by acetic acid [44]. The administration of acetic acid through intraperitoneal (IP) route increases the secretion of inflammatory mediators, namely, bradykinin, serotonin, substance P, histamine and prostaglandins which cause the constrictions of abdomen by mitogen-activated protein (MAP) kinase and microglia [45,46]. After IP administration of acetic acid, an increased level of prostaglandin synthesis was observed as well as the PGE2α and PGF2α [47,48]. In the acetic acid-induced writhing test for antinociceptive activity test, the extract of *P. calocarpa* showed a significant decreased in writhing in a dose dependent manner. The 400 mg/kg dose of MEPC exhibited a significant reduction of writhing (44.12%) than the dose 200 mg/kg (24.44%), whereas the standard drug diclofenac sodium exhibited 63.74% inhibition of writhing, as shown in Figure 4.

The biphasic model of formalin-induced licking test was used in the response of nociception, whereas the early phase is short in duration (neurogenic pain) and the late phase is of long duration (15–30 min) with the inflammatory response. The early phase nociception is due to the activation of delta fibers (A and C), which caused pain intervened by the secretion of substance P (SP) [49,50]. The late phase with the inflammatory response is characterized by the release of inflammatory mediators and excitatory amino acid neurotransmitters [51,52]. Here, the antinociceptive activity of methanol extract of *P. calocarpa* was evaluated by formalin-induced licking test that showed a significant (*p* < 0.001) reduction of nociception in a dose dependent manner in both early and late phase. The 400 mg/kg dose of MEPC exhibited a significant reduction of pain (56.03%) than at 200 mg/kg (38.55%) in the early phase, whereas the standard drug diclofenac sodium exhibited 68.68%. In the late phase, the 400 mg/kg dose of MEPC and standard drug diclofenac sodium exhibited reduction of pain by 53.39% and 63.16%, respectively (Table 1).

### 2.5. Effect of Methanol Extract of P. calocarpa on Antidiarrheal Activity

**Figure 5:**
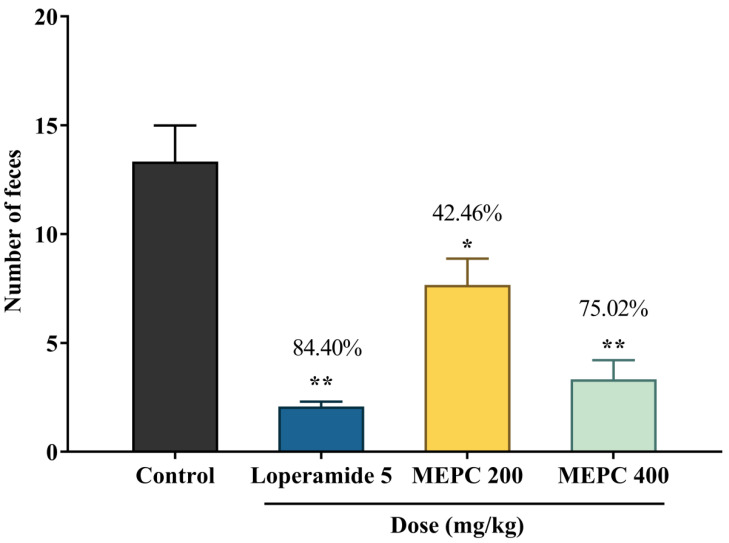
Antidiarrheal activity of methanol extract of *P. calocarpa* (MEPC) and loperamide in castor oil-induced diarrhea in mice. Values represented as mean ± SEM (*n* = 5). * *p* < 0.01 and ** *p* < 0.001 considered as significantly different from the control (Dunnett’s test).

Several pathophysiological conditions cause diarrhea, such as; (i) osmotic diarrhea which is increased luminal osmolarity; (ii) secretory diarrhea which is increased secretion of electrolytes, (iii) reduced electrolytes absorption and (iv) irregular intestinal motility [53]. Castor oil has been commonly used to induce diarrhea for antidiarrheal activity studies, where it secretes ricinoleic acid. Ricinoleic acid is responsible for causing diarrhea, whereas the nitric acid in castor oil is also responsible for the diarrheal effect [54]. The mechanism of ricinoleic acid to initiates diarrhea by GI mucosa irritation led to the secretion of prostaglandin, which stimulates intestinal motility and electrolyte discharge [55]. Hence, the antidiarrheal effect of the medicinal plants may be due to the effects that resist the actions of castor oil to induce diarrhea. The antidiarrheal activity of MEPC in the castor oil-induced diarrheal test demonstrated a significant reduction of defecation in a dose-dependent manner where 400 mg/kg dose of MEPC exhibited a significant (*p* < 0.001) reduction of defecation (75.02%) than the dose 200 mg/kg (42.46%). The standard drug loperamide demonstrated 84.40% inhibition of defecation, as shown in Figure 5.

Moreover, the antidiarrheal activity of MEPC on intestinal motility by charcoal marker indicated a significant reduction in peristalsis index. The 400 mg/kg dose of MEPC exhibited peristalsis index (45.26% ± 7.71%), which was almost similar to the standard drug loperamide (43.05% ± 2.79%). MEPC (200 and 400 mg/kg) demonstrated 22.14% and 43.51% reduction of intestinal motility, whereas the loperamide showed 48.09% reduction of intestinal motility. The result is shown in Table 2. The MEPC reported to have flavonoid and phenol in their semiqualitative phytochemical analysis, where the flavonoids can resist the intestinal motility, secretion of water–electrolytes and intestinal secretion by prostaglandin E2 [56,57]. Therefore, the significant antidiarrheal activity of the MEPC may be probably due to the presence of flavonoids and phenols.

### 2.6. Effect of Methanol Extract of P. calocarpa on Antioxidant Activity

**Figure 6:**
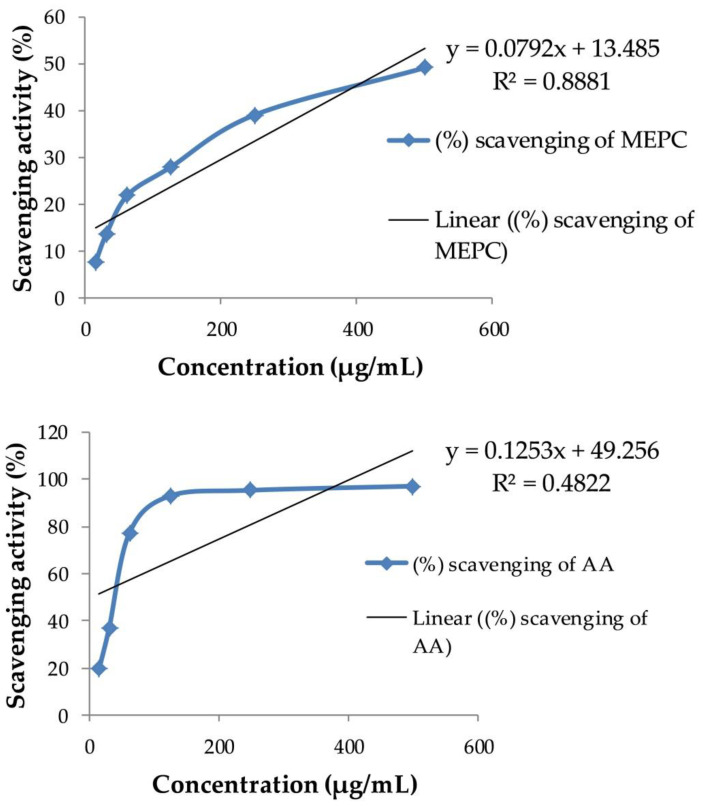
DPPH-scavenging activity of methanol extract of *P. calocarpa* (MEPC) and standard ascorbic acid (AA).

Reactive oxygen and nitrogen species are chemical species that incorporate radical and non-radical oxygen species formed by the decrease of oxygen (H_2_O_2_, HO**^•^**, NO**^•^** and ONOO^¯^) [58]. The source of ROS/RNS is oxidative phosphorylation in mitochondria by the leaking of electron that generates free oxygen radicals [58,59]. Moderate concentrations of ROS play vital roles in numerous biologic processes (cell cycle regulation, phagocytosis, gene expression and cell signaling), whereas excess production can be lead to severe pathologic damages. In particular, increased concentrations of ROS led to OS, which is required to neutralize and detoxify byproducts and biologic system [59,60,61,62]. Inside the body, antioxidants act to inhibit the reactions among free radicals and biologic substances by interrupting the reaction of radical oxidation [63]. Various techniques are utilized to assess the radical-scavenging activity of antioxidants. DPPH-scavenging strategy is a favored technique since it is quick, simple and reliable. DPPH is a steady, synthetic radical that does not break down in water, methanol or ethanol. The free radical-scavenging activity of plant extracts rely upon the capacity of compounds to release hydrogen and the basic conformation of these compounds [64]. In our present study, the DPPH radical-scavenging assay of MEPC and ascorbic acid was presented in Figure 6. The inhibitory concentration (IC_50_) of MEPC was 461.05 µg/mL, whereas for the standard drug ascorbic acid showed 5.94 µg/mL (Table 3). MEPC and ascorbic acid exhibited a concentration-dependent radical-scavenging activity where 49.06% and 97.05% highest scavenging activity was observed by 500 µg/mL dose of ascorbic acid and MEPC, respectively.

Phenolic compounds scavenge free radicals and release hydrogen atoms and electrons to exhibit antioxidant capacity [65] The phenolic substances are effective in intervening neurodegenerative diseases, where the flavonoids reported to studied for preventing the neurodegeneration [66,67]. In quantitative phytochemical analysis, the total phenol content (TPC) of the MEPC was demonstrated as 118.31 ± 1.12 GAE/g of extract which was calculated from the calibration curve (y = 0.0039x + 0.0406; R^2^ = 0.9981). The total flavonoid content (TFC) of the MEPC was found as 100.85 ± 0.97 mg QE/g extract (y = 0.0102x − 0.0637; R^2^ = 0.9693). The result of TPC and TFC was presented in Table 3.

### 2.7. Effect of Methanol Extract of P. calocarpa on Thrombolytic Activity

**Figure 7:**
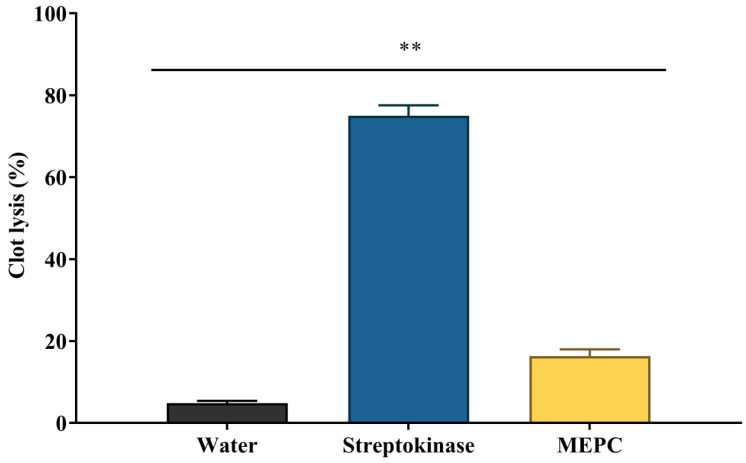
Thrombolytic activity of methanol extract of *P. calocarpa* (MEPC) and streptokinase in comparison with the water. Values represented as mean ± SEM (*n* = 10). ** *p* < 0.001 considered as significantly different from the control (Dunnett’s test).

The evaluation of thrombolytic activity is a significant finding, which may be implicated in cardiovascular-related problems in health. Thrombolysis is the breakdown of clots in blood by drugs where the plasmin (fibrinolytic agent) breakdown the clot that contained as fibrinogen and fibrin [68]. The streptokinase (SK) is additionally used to break down plasminogen to plasmin. This kind of drugs is developed from the Streptococcus species, or, utilizing the recombinant biotechnology [69]. However, there are a few thrombolytic drugs available with lesser side effects which lead the researcher to develop new drugs from natural sources [70]. There are few drugs developed from the natural sources which exert thrombolytic effects, for example, *A. sativum* [71] *F. arabica* [72] and *C. sinensis* [73]. In the present study, the MEPC and streptokinase showed 16.33% and 75% (*p* < 0.001) clot lysis activity, respectively in comparison with the control (4.84%), as shown in Figure 7.

### 2.8. Effect of Methanol Extract of P. calocarpa on Cytotoxicity Activity

**Figure 8:**
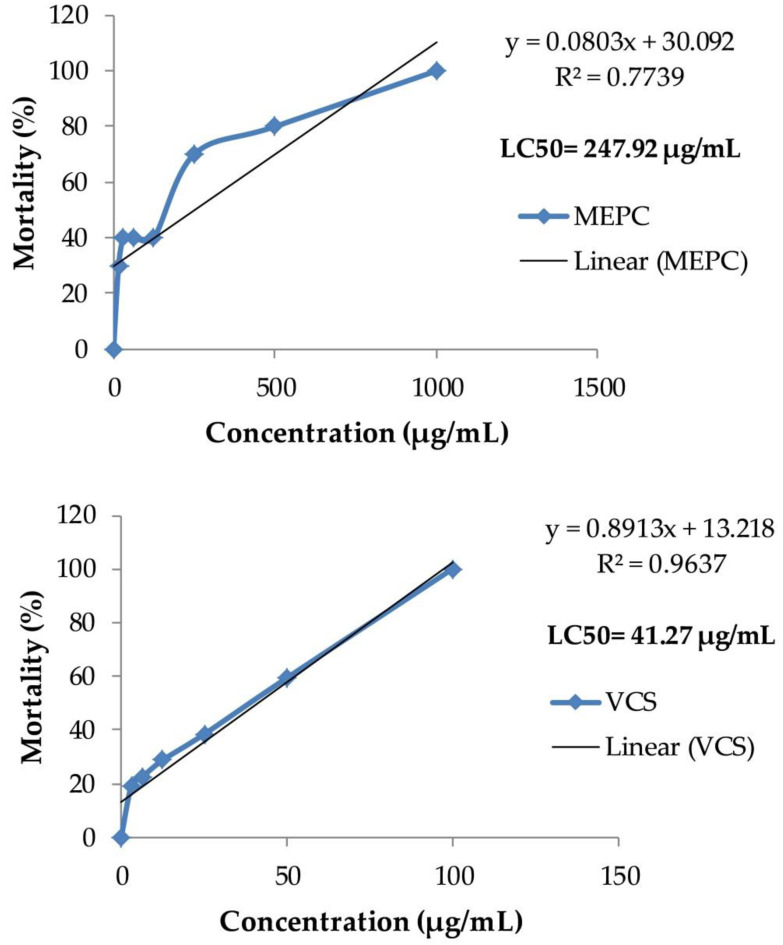
Brine shrimp lethality bioassay of methanol extract of *P. calocarpa* (MEPC) and vincristine sulfate (VCS). The LC_50_, values of samples were extrapolated using this data.

The brine shrimp lethality bioassay is a quick, cheap and basic protocol for testing the plant extracts for assessing cytotoxic and antitumor effects [74]. Commonly, an ideal biologic response is not because of one substance rather a combination of bioactive substance from plant parts. Hence, crude plant extract required to analyze the biologic effect [74]. Generally, the greater the LC_50_ value, the less toxic the substance of the extract is and vice versa. A crude plant extract with a LC_50_ value of less than 100 µg/mL is considered as highly toxic, 100–500 µg/mL is moderately toxic, 500–1000 µg/mL is weakly toxic and above 1000 µg/mL is mentioned as safe or nontoxic [75]. The cytotoxicity activity of MEPC was presented in Figure 8, where a concentration-dependent lethality was observed for both MEPC and standard drug vincristine sulfate. The MEPC showed moderate toxicity with LC_50_ value of 247.92 μg/mL, whereas the LC_50_ value of vincristine sulfate (VCS) was 41.27 µg/mL. This moderate toxicity of MEPC may be due to the presence of alkaloid in the phytochemicals [76].

### 2.9. Molecular Docking Study

The process of drug discovery is complex and incorporates an interdisciplinary exertion for designing a therapeutically effective and commercially possible drug. The computer plays an important role in pharmaceutical and medicinal research [77]. Computer-aided drug design (CADD) developed as a productive method for finding potential lead compounds and for helping the advancements of potential medications for a wide range of illnesses [78]. Today, various computational methodologies are being utilized to find potential lead compounds from immense compound libraries. Molecular docking has a lack of confidence on the scoring that provides after interaction of protein and ligand, but it is a topic of interest for the researcher. To ensure the binding affinity of the protein and ligand, high-resolution X-ray, QSAR or molecular dynamics study required [79]. In our present study, the isolated compound psychotriasine from the *P. calocarpa* subjected to molecular docking study shown in Table 4 and the docking figures are shown in Figures S1–S8.

Molecular docking study for anxiolytic and antidepressant activity of psychotriasine revealed −3.359 kcal/mol and −6.548 kcal/mol docking score against the potassium channel receptor (PDB: 4UUJ) and the human serotonin receptor (PDB: 5I6X), respectively, whereas the reference drug diazepam and fluoxetine produced (−2.875 kcal/mol and −8.576 kcal/mol, respectively). For antinociceptive activity, psychotriasine exposed −7.81 kcal/mol and −5.308 kcal/mol docking score against COX-1 (PDB: 2OYE) and COX-2 (PDB: 3HS5) enzymes, respectively. However, the standard drug diazepam did not pose any interaction against the COX-1 (PDB: 2OYE) and COX-2 (PDB: 3HS5) enzymes. For antidiarrheal docking analysis psychotriasine significantly presented −5.811 kcal/mol and −8.826 kcal/mol against 5-HT3 receptor (PDB: 5AIN) and M3 muscarinic acetylcholine receptor (PDB: 4U14), respectively. The present study showed that psychotriasine demonstrated −4.053 kcal/mol docking score against urate oxidase (PDB: 1R4U) for antioxidant activity, whereas standard drug ascorbic acid exhibited −4.789 kcal/mol docking score. On the other hand, psychotriasine was found to possess higher docking score than the standard, which was −5.817 kcal/mol against human tissue plasminogen activator (PDB: 1A5H) for thrombolytic activity. The result of cytotoxic docking study, psychotriasine displayed −5.18 kcal/mol against human estrogen receptor (PDB: 3ERT). The binding interactions of psychotriasine are shown in Table 5.

### 2.10. ADME/T and Toxicological Properties

ADME/T profiling plays an important role in the discovery and development process of the drug. A drug should not only have satisfactory efficacy against the possible therapeutic target yet additionally needed to show the suitable ADME/T properties. Several ADME/T profiling in silico methods are developed, but it is as yet difficult to assess the drug-like behavior of compounds due to several ruling for ADME/T properties. In our study, the isolated compound psychotriasine from the *P. calocarpa* subjected to the ADME/T and toxicological properties analysis by following the Lipinski’s rule of five [80] and Veber rules [81]. The compound psychotriasine did not violate any rules of Lipinski’s and Veber, which indicate the possible oral bioactivity of the compounds. Additionally, psychotriasine exhibited non-ames toxic, noncarcinogens, acute oral toxicity (III) and a weak rat acute toxicity. The results of ADME/T and toxicological properties were presented in Table 6.

## 3. Materials and Methods

### 3.1. Chemicals

Diazepam, fluoxetine, loperamide and diclofenac sodium were acquired from Square Pharmaceuticals, Ltd. (Dhaka, Bangladesh), while streptokinase and Vincristine sulfate (2 mg/vial) was purchased from Beacon Pharmaceuticals, Ltd. Bangladesh. Aluminum chloride, 1, 1-diphenyl2-picrylhydrazyl (DPPH), gallic acid and Folin–Ciocâlteu reagent (FCR) were procured from Sigma-Aldrich (Bangalore, India). Ascorbic acid and quercetin were procured from BDH Chemicals, Ltd. (Poole, UK). Absorbance was taken by UV-vis spectrophotometer (UVmini-1240, Shimadzu, Japan). The other chemicals were purchased in analytical grades from local trader through Taj Scientific, Ltd. (Chittagong, Bangladesh)

### 3.2. Animals

Swiss albino mice of either sex weighing about 25–35 g (six–seven weeks) were purchased from the Jahangirnagar University, Dhaka −1343, Bangladesh. The mice were familiarized with the standard conditions by maintaining in room temperature 25 ± 2 °C, relative humidity 55–60% with a 12 h light/dark cycle. The animals were provided with proper food pellets and water supply. The study was conducted under the Animal Research: Reporting of In Vivo Experiments (ARRIVE) Guidelines for in vivo study and the Institutional Animal Ethical Committee, Department of Pharmacy, International Islamic University Chittagong, Bangladesh approved the study protocol under the reference number Pharm/P&D/138/13-19 [82].

### 3.3. Collection and Preparation of Methanol Extract

The leaves of *P. calocarpa* were collected from the Hajarikhil Hill tract, Chittagong, Bangladesh in the month of February 2019, which was identified by Professor Dr Shaikh Bokhtear Uddin, Department of Botany, University of Chittagong, Bangladesh. The leaves were collected in fresh condition from the shrubs (1–1.5 m). At first, the leaves were washed with water and later shaded dried at 55–60 °C for seven days. The dried leaves were ground into the coarse powder (1.18 mm) by a grinder (NOWAKE, Japan) and soaked the powder (500 g) in methanol (2 L) for 15 days with occasional shaking and stirring. The extraction process was done in a standard laboratory condition (27 ± 2 °C). After 15 days, the filtration was done using Whatman #1 filter paper and the final filtrate was evaporated in the water bath at 45 °C. Lastly, the obtained sticky semi-solid part of the methanol extract of *P. calocarpa* leaves (MEPC) was preserved in 4 °C.

### 3.4. Semiqualitative Phytochemical Screening

The semiqualitative phytochemical analysis of the methanol extract of *P. calocarpa* leaves was carried out by the standard methodology for testing the alkaloid, glycosides, tannins, saponins, resins, carbohydrate, flavonoid, phenols, terpenoids, quinones and proteins [83,84,85], which briefly presented in the Supplementary Section.

### 3.5. Experimental Design

In this study, five Swiss albino mice of either sex were used in each group (control, standard and MEPC). The MEPC group was administrated 200 and 400 mg/kg accordance with their body weight (BW), whereas the control group was administrated 1% Tween-80 in water (10 mL/kg, BW). Diazepam (1 mg/kg, BW, IP) was used for the anxiolytic (elevated plus maze test, hole–board test and light and dark test) and locomotor activity (open field test), while fluoxetine (10 mg/kg, BW, IP) was used for antidepressant activity (forced swim test and tail suspension test). Diclofenac sodium (10 mg/kg BW, IP) and loperamide (5 mg/kg BW, IP) were used for testing the antinociceptive (acetic acid-induced writhing test and formalin-induced licking test) and antidiarrheal activity (castor oil-induced diarrhea and intestinal motility test), respectively.

### 3.6. Anxiolytic Activity

#### 3.6.1. Elevated Plus Maze (EPM) Test

The elevated plus maze (EPM) test was used to examine the anxiolytic activity of plant extract MEPC in mice [86]. The apparatus assembled by two open arms (5 × 10 cm^2^) and two closed arms (5 × 10 × 15 cm^3^), which was elevated from the floor in the height of 40 cm. The experiment of each group was followed, as described in Section 3.5. Thirty minutes after the treatment, each treated mice was placed in the middle point of the EPM apparatus facing towards the closed arms and recorded for 5 min. The percentage of entries and time spent was calculated by the following equation.

(1) $$\%\;\mathrm{of}\;\mathrm{entries}\;\mathrm{in}\;\mathrm{the}\;\mathrm{open}\;\mathrm{arm}=\frac{\mathrm{No}.\;\mathrm{of}\;\mathrm{entries}\;\mathrm{in}\;\mathrm{the}\;\mathrm{open}\;\mathrm{arm}}{\mathrm{No}.\;\mathrm{of}\;\mathrm{entries}\;\mathrm{in}\;\mathrm{the}\;\mathrm{open}\;\mathrm{arm}+\mathrm{No}.\;\mathrm{of}\;\mathrm{entries}\;\mathrm{in}\;\mathrm{the}\;\mathrm{closed}\;\mathrm{arm}}\;\times \;100$$

(2) $$\%\;\mathrm{of}\;\mathrm{time}\;\mathrm{spent}\;\mathrm{in}\;\mathrm{the}\;\mathrm{open}\;\mathrm{arm}=\frac{\mathrm{Time}\;\mathrm{spent}\;\mathrm{in}\;\mathrm{the}\;\mathrm{open}\;\mathrm{arm}}{\mathrm{Time}\;\mathrm{spent}\;\mathrm{in}\;\mathrm{the}\;\mathrm{open}\;\mathrm{arm}+\mathrm{Time}\;\mathrm{spent}\;\mathrm{in}\;\mathrm{the}\;\mathrm{closed}\;\mathrm{arm}}\;\times \;100$$

#### 3.6.2. Hole-Board Test (HBT)

The anxiolytic activity was assessed by the hole–board test, which consists of 16 evenly distributed holes 3 cm in diameter in a wooden space (40 × 40 × 25 cm^3^). The apparatus elevated from the floor at the height of 25 cm. The dosing of each group was followed, as described in Section 3.5. After thirty minutes of the treatment, each treated mice individually was placed in the center of the board. The animals were allowed to move freely and the movement recorded for 5 min [87].

#### 3.6.3. Light and Dark Box Test (LDT)

The anxiolytic activity of MEPC was evaluated by the light and dark test, which comprised of the four-sided box (46 × 27 × 30 cm^3^). The box divided by 18 × 27 cm^2^ into a small area and 27 × 27 cm^2^ into a large area with a door, which positioned in the center of the dividing wall. The dosing of each group was followed, as described in Section 3.5. Thirty minutes after the administration, each treated mice was placed individually in the dark compartment and recorded the time spent in the compartment for 5 min [88].

### 3.7. Locomotor Activity

#### Open Field Test (OFT)

The locomotor activity of MEPC was assessed by the open field test according to the previously described method of Gupta et al. (1971) [89]. A four-sided box with a measurement of 60 × 60 × 60 cm^3^ with 25 equal squares (5 × 5 cm^2^), which alternatively highlighted in black and white. The dosing of each group was followed, as described in Section 3.5. Immediately after the treatment, each mouse was individually placed in the apparatus for 3 min at 0, 30, 60, 90 and 120 min intervals, where the number of squares moved was recorded.

### 3.8. Antidepressant Activity

#### 3.8.1. Forced Swim Test (FST)

The antidepressant activity of MEPC and standard drug fluoxetine was evaluated by forced swimming test by previously described protocol [90]. The FST assessed in a transparent glass beaker (25 × 15 × 25 cm^3^) which occupied with water (25 ± 1 °C) up to 15 cm. The dosing of each group was followed, as mentioned in Section 3.5. Thirty minutes after the administration, each mouse was individually placed in the apparatus for forced swimming. The study conducted for 6 min, where the first 2 min was used for initial adjustment and the last 4 min was considered as the immobility time.

(3) $$\mathrm{Inhibition}\;\left(\%\right)=\frac{A-B}{A}\times 100$$

where, A = immobile time in the control group; B = immobile time in the test group.

#### 3.8.2. Tail Suspension Test (TST)

The antidepressant activity of MEPC and standard drug fluoxetine was evaluated by forced swimming test by the previously described method of Steru et al. 1985 [91]. The treatment of each group was followed, as mentioned in Section 3.5. After the administration, each mice group was individually hanged by the end of the tail (approximately 1 cm) using tape. The study conducted for 6 min, where the first 2 min was used for initial adjustment and the last 4 min was considered as the immobility time.

(4) $$\mathrm{Inhibition}\;\left(\%\right)=\frac{A-B}{A}\times 100$$

where, A = immobile time in the control group; B = immobile time in the test group.

### 3.9. Antinociceptive Activity

#### 3.9.1. Acetic Acid-Induced Writhing Inhibition Test

The acetic acid-induced writhing test was used for the evaluation of the antinociceptive activity of MEPC by the previously described method [92]. The treatment followed the process, as mentioned in Section 3.5. Thirty minutes after the administration, 0.7% acetic acid (*v/v*) was injected in the intraperitoneal route of mice. The total writhing by each mouse was recorded for 15 min. Following equation was used to evaluate the percentage of inhibition:

(5) $$\mathrm{Inhibition}\;\left(\%\right)=\frac{A-B}{A}\times 100$$

where, A = mean number of writhing by control group; B = mean number of writhing by test group.

#### 3.9.2. Formalin-Induced Licking Test

The antinociceptive activity of MEPC was assessed by the formalin-induced licking test, as described by Okokon et al. 2010 [93]. The treatment followed the process, as mentioned in Section 3.5. After 30 min of the treatment, 20 μL of formalin solution (2.5% v/v) was injected into the sub-plantar region (hind paw) of the mice. The licking times were recorded as early phase (0–5 min) and late phase (15–30 min). Following equation was used to evaluate the percentage of inhibition:

(6) $$\mathrm{Inhibition}\;\left(\%\right)=\frac{A-B}{A}\times 100$$

where, A = mean licking time by control group; B = mean licking time by test group.

### 3.10. Antidiarrheal Activity

#### 3.10.1. Castor Oil-Induced Diarrhea

The antidiarrheal activity was evaluated by castor oil-induced diarrhea test following the previously described method [94]. Each mouse was fasted for 24 h before starting the experiment and treatment followed the process as mentioned in Section 3.5. After one hour, 0.5 mL castor oil-induced orally and placed each mouse individually in a separate cage with blotting paper. The total number of feces counted for 4 h and replaced the blotting paper in every one hour after. The following equation was followed for the calculation of percentage inhibition of defecation:

(7) $$\mathrm{Inhibition}\;\left(\%\right)=\frac{A-B}{A}\times 100$$

where, A = average feces number of the control group; B = average feces number of the test group.

#### 3.10.2. Intestinal Motility by Charcoal Marker

The Intestinal motility by the charcoal marker for the antidiarrheal study of MEPC was evaluated by Mascolo et al. 1994 [95]. Each mouse was fasted for 24 h before starting the experiment and treatment followed the process as mentioned in Section 3.5. After one hour, each mouse received 1 mL charcoal solution (10% charcoal in 5% gum acacia). One hour after charcoal administration, each mouse was sacrificed and measured the distance traveled by the charcoal marker and total length of the intestine. The following equation was followed for the calculation of the percentage of inhibition and peristalsis index:

(8) $$\mathrm{Inhibition}\;\left(\%\right)=\frac{A-B}{A}\times 100$$

where, A = Distance travel by the control (cm); B = Distance travel by the test groups (cm)

(9) $$\mathrm{Peristalsis}\;\mathrm{index}=\frac{\mathrm{Distance}\;\mathrm{travel}\;\mathrm{by}\;\mathrm{the}\;\mathrm{charcoal}\;\mathrm{solution}}{\mathrm{Total}\;\mathrm{length}\;\mathrm{of}\;\mathrm{the}\;\mathrm{small}\;\mathrm{intestine}}\times 100$$

### 3.11. Antioxidant Activity

#### 3.11.1. DPPH Free Radical-Scavenging Assay

The antioxidant activity of MEPC was evaluated by DPPH free radical-scavenging assay as described by Braca et al. 2001 [96]. In this experiment, a serially diluted concentration (15.625–500 µg/mL) was followed for the MEPC and standard antioxidant ascorbic acid. Three milliliters of 0.004% DPPH solution (4 mg DPPH in 100 mL of 95% methanol) was mixed with serially diluted concentration (0.1 mL) and allowed to incubate for 30 min at room temperature (25 °C) in the dark condition. The absorbance was read at 517 nm. Additionally, the study followed in triplicate manner.

(10) $$\mathrm{Scavenging}\;\mathrm{effect}\;\left(\%\right)=\frac{\mathrm{absorbance}\;\mathrm{of}\;\mathrm{the}\;\mathrm{control}-\mathrm{absorbance}\;\mathrm{of}\;\mathrm{the}\;\mathrm{test}\;\mathrm{sample}}{\mathrm{absorbance}\;\mathrm{of}\;\mathrm{the}\;\mathrm{control}}$$

#### 3.11.2. Total Phenol Content (TPC)

The semi-quantitative phytochemical analysis of total phenol content (TPC) of the MEPC was assessed by Reza et al. 2018 [97]. Here, 2.5 mL of 10% Folin–Ciocâlteu reagent and 2.5 mL of Na_2_CO_3_ (20%), respectively was added to the 500-μg/mL extract and the solution was filled up to 10 mL by distilled water (D.W.). The solution was allowed to incubate for 20 min at 25 °C and the absorbance was read at 765 nm. Additionally, the study followed in triplicate manner. The TPC of the MEPC measured from the calibration curve of gallic acid solution (standard), whereas the TPC was expressed in milligrams of gallic acid equivalents (GAE) per gram of extracts (mg GAE/g extract).

#### 3.11.3. Total Flavonoid Content (TFC)

The total flavonoid content (TFC) of the MEPC was assessed, according to Reza et al. 2018 [97]. Here, 500 μg/mL extract mixed with methanol (1.5 mL), 10% AlCl_3_ (0.1 mL), 1 M CH_3_COOK (0.1 mL) and distilled water (2.8 mL), respectively. Then, the solution was allowed to incubate for 30 min at 25 °C and the absorbance was taken at 415 nm. Additionally, the study followed in triplicate manner. The TFC of the MEPC measured from the calibration curve of quercetin solution (standard), whereas the TFC was expressed in milligrams of quercetin equivalents (QE) per gram of extracts (mg QE/g extract).

### 3.12. Thrombolytic Activity

The thrombolytic activity of MEPC leaves was evaluated by human blood clot lysis activity as described by Prasad et al. 2006 [98]. In total ten male- female 20–25 years (*n* = 10) healthy volunteer (non-smokers, no history of medication in last two week) were used for this study and 3 mL of blood withdrawn from the venous and 0.5 mL per Eppendorf distributed. The Eppendorf was previously weight. Then, the Eppendorf with blood was incubated for 45 min at 37 °C to form the clot. After forming a clot, the obtained serum in the clot was absolutely removed and again reweighed each Eppendorf. Briefly, 100 μL of MEPC extract (10 mg/mL) added to each Eppendorf. Similar protocols were followed for the standard drug streptokinase (100 μL) and control distilled water (100 μL). All the Eppendorf was allowed to incubate for 90 min at 37 °C and observed clot lysis. Again, the released fluid after incubation was removed and reweighed [68].

Clot lysis (%) = (weight of clot after remove of fluid/clot weight) × 100(11)

### 3.13. Brine Shrimp Lethality Bioassay

The brine shrimp lethality bioassay was used to evaluate the cytotoxic activity of MEPC as depicted by Meyer et al., 1982 [99]. The cytotoxicity of MEPC evaluated using simple organism *Artemia salina*. Here, 2.5 g brine shrimp cyst added in the artificial seawater (3.8% NaCl solution/L, *w/v*) in a beaker (1 L) by maintaining the temperature at 25 ± 1 °C and pH (8.0) with proper light intensity (60 W). The distance between light and brine shrimp beaker was 9 cm. The shrimp eggs hatched for 48 h for maturing the shrimp. In this experiment, a serially diluted concentration (15.625–1000 µg/mL) was followed for the MEPC, while 3.13–100 μg/mL was used for vincristine sulfate. Finally, all test tubes containing 5 mL freshly prepared artificial seawater, where 10 nauplii transferred carefully. Additionally, the study followed in triplicate manner. After 24 h, all test tubes inspected by an amplifying glass and the number of living nauplii in each test tube was observed and recorded [68].

% of mortality = (N_1_/N_0_) × 100 (12)

where, N_0_ = the number of nauplii taken; N_1_ = the number of nauplii dead.

### 3.14. In Silico Study

#### 3.14.1. Molecular Docking Analysis

The isolated compounds from the leaves of *P. calocarpa* by NMR analysis [25] was subjected for molecular docking study by following the protocol of Sastry et al. 2013, which briefly explained in Tareq, 2019 [100,101]. The 3D structure of receptors/enzymes were retrieved from the Protein Data Bank (https://www.rcsb.org/structure): potassium channel KCSA-FAB receptor (PDB ID: 4UUJ), structure of the ts3 human serotonin transporter receptor (PDB ID: 5I6X), cyclooxygenase-1 (PDB ID: 2OYE), cyclooxygenase-2 (PDB ID: 3HS5), 5-HT3 receptor (PDB: 5AIN), M3 muscarinic acetylcholine receptor (PDB: 4U14), urate oxidase (PDB: 1R4U), human tissue plasminogen activator (PDB: 1A5H) and human estrogen receptor (PDB: 3ERT) [102]. Molecular docking study was analyzed by Schrödinger Mestro (v11.1).

#### 3.14.2. ADME/T and Toxicological Properties Analysis

The absorption, distribution, metabolism, excretion and toxicity (ADME/T) analysis of psychotriasine was analyzed by the Lipinski’s rules [80] and Veber’s rules [81], whereas the toxicological properties assessed by admetSAR (Online tool). The ADME/T analysis was assessed by SwissADME (http://www.swissadme.ch/) and QikProp (Schrödinger v11.1) [103].

### 3.15. Statistical Analysis

Values represented as mean ± S.E.M (standard error mean). * *p* < 0.01 and ** *p* < 0.001 considered as significantly different from the control; one-way ANOVA (Dunnett’s test) was carried out using GraphPad Prism version 8.4., whereas two-way ANOVA with repeated measures was followed for the open filed test.

## 4. Conclusions

The present pharmacological activity study revealed that MEPC may have significant neuropharmacological effects due to secondary metabolites, namely alkaloids and flavonoids effective in neurotransmission systems. Additionally, MEPC had an antinociceptive activity, which may possibly suppress the inflammatory mediators. A significant and dose-dependent antidiarrheal activity observed in different models. In addition, a strong antioxidant activity was observed in phytochemical and free radical-scavenging analysis, which possibly able to inhibit the oxidative related disorders. Furthermore, the MEPC showed a moderate toxicity level with a significant clot lysis activity. Computational study of isolated compound psychotriasine revealed a favorable binding affinity towards the different receptors with a good ADME/T and toxicological profile. A further advanced mechanistic analysis is required to ensure the responsible compounds for these activities.

## Figures and Tables

**Table 1:** Antinociceptive activity of methanol extract of *P. calocarpa* (MEPC) in formalin-induced licking test in mice.

Treatment	Early Phase (sec)	Inhibition (%)	Late Phase (sec)	Inhibition (%)
Control	55.33 ± 4.33	-	44.33 ± 0.33	-
Diclofenac sodium	17.33 ± 0.33 **	68.68	16.33 ± 0.33 **	63.16
MEPC 200	34.0 ± 3.21 **	38.55	26.33 ± 1.76 **	40.60
MEPC 400	24.33 ± 1.45 **	56.03	20.66 ± 1.20 **	53.39

Values represented as mean ± SEM (*n* = 5). ** *p* < 0.001 considered as significantly different from the control (Dunnett’s test).

**Table 2:** Antidiarrheal activity of methanol extract of *P. calocarpa* (MEPC) on intestinal motility in mice by charcoal as a marker.

Treatment	Total Length of Intestine (cm)	Distance Travel by Charcoal (cm)	Peristalsis Index (%)	% Inhibition
Control	50.33 ± 0.33	43.67 ± 2.91	86.69 ± 5.23	-
Loperamide	52.67 ± 1.20	22.67 ± 1.45 **	43.05 ± 2.79 **	48.09
MEPC 200	60.0 ± 3.21 *	34.0 ± 1.53	56.72 ± 0.73 *	22.14
MEPC 400	54.33 ± 0.66	24.67 ± 4.41 **	45.26 ± 7.71 **	43.51

Values represented as mean ± SEM (*n* = 5). * *p* < 0.01 and ** *p* < 0.001 considered as significantly different from the control (Dunnett’s test).

**Table 3:** Antioxidant activity by total phenol content, total flavonoid content of methanol extract of *P. calocarpa* leaves with IC_50_ value.

Treatment	Total Phenol Content (mg GAE/g EXTRACT)	Total Flavonoid Content (mg QE/g Extract)	IC_50_ (µg/mL)
**MEPC**	118.31 ± 1.12	100.85 ± 0.97	461.05
**Ascorbic acid**	-	-	5.94

MEPC—methanol extract of *Psychotria calocarpa* leaves.

**Table 4:** Docking score of the psychotriasine isolated from the *P. calocarpa*.

Compounds	Docking Score(kcal/mol)								
	4UUJ	5I6X	2OYE	3HS5	5AIN	4U14	1R4U	1A5H	3ERT
Psychotriasine	−3.359	−6.548	−7.81	−5.308	−5.811	-8.826	−4.053	−5.817	−5.18
Standard drugs	−2.875	−8.576	-	-	-	−6.429	−4.789	−5.704	-
(DZ/FX/DS/LA/AA/SK/VCS)									

DZ—diazepam; FX-fluoxetine; DS—diclofenac sodium; LA—loperamide; AA—ascorbic acid; SK—streptokinase; VCS—vincristine sulfate.

**Table 5:** Binding interaction of psychotriasine with different receptors.

Proteins	Hydrogen Bond Interactions		Hydrophobic Interactions	
	Amino Acid Residue	Distance (Å)	Amino Acid Residue	Distance (Å)
4UUJ	PO-4113	2.71	LEU-86	4.81
			-	5.07
			-	4.33
			ARG-89	6.33
	GLU-493	5.12	ASP-98	6.47
5I6X	ASP-98	5.43	TYR-98	6.01
	SER-438	3.62	ILE-172	4.86
	TYR-335	6.16	GLY-526	5.42
2OYE	SER-530	5.33	-	5.45
	LEU-384	2.93	VAL-349	4.44
3HS5	HIS-207	5.13	HIS-388	8.37
	GLN-203	5.02	LEU-294	5.24
5AIN	TYR-91	4.99	CYS-189	4.92
			CYS-188	5.56
			TYR-53	6.58
4U14	ALA-238	4.54	ALA-238	6.05
1R4U	THR-168	3.16	LEU-170	4.95
1A5H	GLY-216	4.59	GLN-192	4.41
			TYR-99	6.32
3ERT	LEU-346	4.68	TRP-383	6.33
			-	7.29
			ASP-351	6.67
			LEU-525	4.16
			MET-522	6.94

**Table 6:** ADME/T and toxicological properties of psychotriasine isolated from the *P. calocarpa.*

ADME/T and Toxicological Properties of Psychotriasine	
Parameters	Values
molecular weight (<500 g/mol)	346.47
Hydrogen bond donor (<5)	2
Hydrogen bond acceptor (<10)	2
High lipophilicity (LogP, <5)	3.02
Rotatable bond (≤10)	4
Topological polar surface area (≤140)	32.23 Å^2^
Ames toxicity	Non AMES toxic
Carcinogens	Noncarcinogens
Acute oral toxicity	III
Rat acute toxicity (LD_50_, mol/kg)	2.7118
Rule of five violations	0

## Supplementary Materials

The following are available online at https://www.mdpi.com/1424-8247/13/8/183/s1, Table S1: Semiqualitative phytochemical screening of *P. calocarpa* leaves; Figure S1: 3D and 2D interactions of psychotriasine (A) and diazepam (B) with the potassium channel receptor (PDB: 4UUJ) for anxiolytic activity; Figure S2: 3D and 2D interactions of psychotriasine (A) and fluoxetine (B) with the human serotonin receptor (PDB: 5I6X) for antidepressant activity; Figure S3: 3D and 2D interactions of psychotriasine with COX-1 (PDB: 2OYE, A) and COX-2(PDB: 3HS5, B)enzyme for antinociceptive activity; Figure S4: 3D and 2D interactions of psychotriasine with 5-HT3 receptor (PDB: 5AIN) for antidiarrheal activity; Figure S5: 3D and 2D interactions of psychotriasine (A) and loperamide (B) with M3 muscarinic acetylcholine receptor (PDB: 4U14) for antidiarrheal activity; Figure S6: 3D and 2D interactions of psychotriasine (A) and Ascorbic acid (B) with the urate oxidase (PDB: 1R4U) for antioxidant activity; Figure S7: 3D and 2D interactions of psychotriasine (A) and streptokinase (B) with the human tissue plasminogen activator (PDB: 1A5H) for thrombolytic activity; Figure S8: 3D and 2D interactions of psychotriasine with the human estrogen receptor (PDB: 3ERT) for cytotoxic activity.

## Abbreviations

MEPC—methanol extract of *Psychotria calocarpa* leaves; OS—oxidative stress; NMR—nuclear magnetic resonance; ADME/T—absorption, distribution, metabolism, excretion and toxicity; IP—intraperitoneal; BW—body weight; PDB—protein data bank; SEM—standard error mean; ANOVA—one-way analysis of variance.
