# Supplementary material for: Deciphering the Pharmacological Properties of Methanol Extract of Psychotria calocarpa Leaves by In Vivo, In Vitro and In Silico Approaches
Source: Pharmaceuticals (Basel). 2020 Aug 6;13(8):183. doi: 10.3390/ph13080183 (PMC7463710; doi:10.3390/ph13080183)
Supplement: Supplementary file 1 [file pharmaceuticals-13-00183-s001.pdf]

Supplementary Materials

Deciphering the pharmacological properties of methanol extract of *Psychotria calocarpa* leaves by in vivo, in vitro and in silico approaches

Tahmina Akter Bristy [1,†], Niloy Barua [1,†], Abu Montakim Tareq [1,†], Shahenur Alam Sakib [2], Saida Tasnim Etu [1], Kamrul Hasan Chowdhury [1], Mifta Ahmed Jyoti [1], Md. Arfin Ibn Aziz [1], A.S.M. Ali Reza [1], Elisabetta Caiazzo [3], Barbara Romano [3], Syed Mohammed Tareq [1,*], Talha Bin Emran [4,*] and Raffaele Capasso [5,*]

[1] Department of Pharmacy, International Islamic University Chittagong, Kumira, Chittagong 4318, Bangladesh; tahminabristy4@gmail.com (T.A.B.); niloybaruaniloy@gmail.com (N.B.); montakim0.abu@gmail.com (A.M.T.); saidatasnim96@gmail.com (S.T.E.); kamrulhasan73132@gmail.com (K.H.C.); mifta_ahmed@yahoo.com (M.A.J.); arfinibnaziz151085@gmail.com (M.A.I.A.); alirezaru@gmail.com (A.S.M.A.R.)

[2] Department of Theoretical and Computational Chemistry, University of Dhaka, Dhaka 1000, Bangladesh; sakibhasaniiuc@gmail.com

[3] Department of Pharmacy, School of Medicine, University of Naples Federico II via Domenico Montesano, Naples 49 80131, Italy; elisabetta.caiazzo@unina.it (E.C.); barbara.romano@unina.it (B.R.)

[4] Department of Pharmacy, BGC Trust University Bangladesh, Chittagong 4381, Bangladesh

[5] Department of Agricultural Sciences, University of Naples Federico II, Portici 80055, Italy

† These authors contributed equally to this work

* Correspondence: mail2babor@gmail.com (S.M.T.); talhabmb@bgctub.ac.bd (T.B.E.); rafcapas@unina.it (R.C.); Tel.: +88-01711-170994 (S.M.T.); +88-01819-942214 (T.B.E.); +39-081-678664 (R.C.)

Table S1: Semi-qualitative phytochemical screening of *P. calocarpa* leaves.

Test Name	Results
Alkaloid	+
Glycosides	+
Tannins	+
Saponins	+
Resins	+
Carbohydrate	−
Flavonoid	+
Phenols	+
Terpenoids	−
Quinones	−
Proteins	−

1. Semi-qualitative Phytochemical Screening

The semi-qualitative phytochemical analysis of the methanol extract of *P. calocarpa* leaves was carried out by the standard methodology for testing the alkaloid, glycosides, tannins, saponins, resins, carbohydrate, flavonoid, phenols, terpenoids, quinones, and proteins [1-3].

1.1. Test for Alkaloids

Two milliliters of extract solution were added with the 2-3 drops of Mayer's reagent, whereas the white precipitates considered as the presence of alkaloids.

1.2. Test for Glycosides

The Borntrager's methodology was followed, whereas 2 mL of extract solution were added with 3 mL of chloroform and shaken well. After shaking, the layer of chloroform was separated and added ammonia solution (10%), whereas the pink color indicates the presence of glycosides.

1.3. Test for Tannins

Five milliliters of extract solution were added with the few drops of ferric chloride solution (5%), whereas the dark green color considered as the presence of tannins.

1.4. Test for Saponins

Three milliliters of extract solution was added with the 10 mL of distilled water (D.W.) in a test tube. The solution was shaken vigorously for five minutes and then allowed to stand still for thirty minutes to form frothing. This frothing indicates the presence of saponins.

1.5. Test for Resins

One milliliter of extract solution were added with the few mL of $C_4H_6O_3$ and 1 mL of conc. H_2SO_4 in a test tube, whereas the conversation of orange to yellow color indicated the presence of resins.

1.6. Test for carbohydrate

The Benedict's methodology was followed, whereas 0.5 mL of extract solution was added with 0.5 mL of Benedict's solution and heated in water bath for 2 min. The red precipitates indicate the presence of carbohydrates.

1.7. Test for Flavonoid

One milliliter of extract solution was added with the few mL of lead acetate (10%) in a test tube, whereas the yellow precipitates indicated the presence of flavonoids.

1.8. Test for Phenols

Five milliliter of extract solution was added with the 3 mL of lead acetate (10%) in a test tube and mixed very gently, whereas the white precipitates indicated the presence of phenols.

1.9. Test for Terpenoids

Three milliliter of extract solution was added with the 1 mL of chloroform and 2 mL of conc. H_2SO_4 in a test tube, whereas the reddish brown color indicated the presence of terpenoids.

1.10. Test for Quinones

One milliliter of extract solution was added with the few mL of alcoholic Potassium hydroxide (KOH) in a test tube, whereas the colour change from red to blue indicated the presence of quinones.

1.11. Test for Proteins

Two milliliter of extract solution was added with the two mL of water and few drops of conc. HNO_3 in a test tube, whereas the yellow color indicated the presence of proteins.

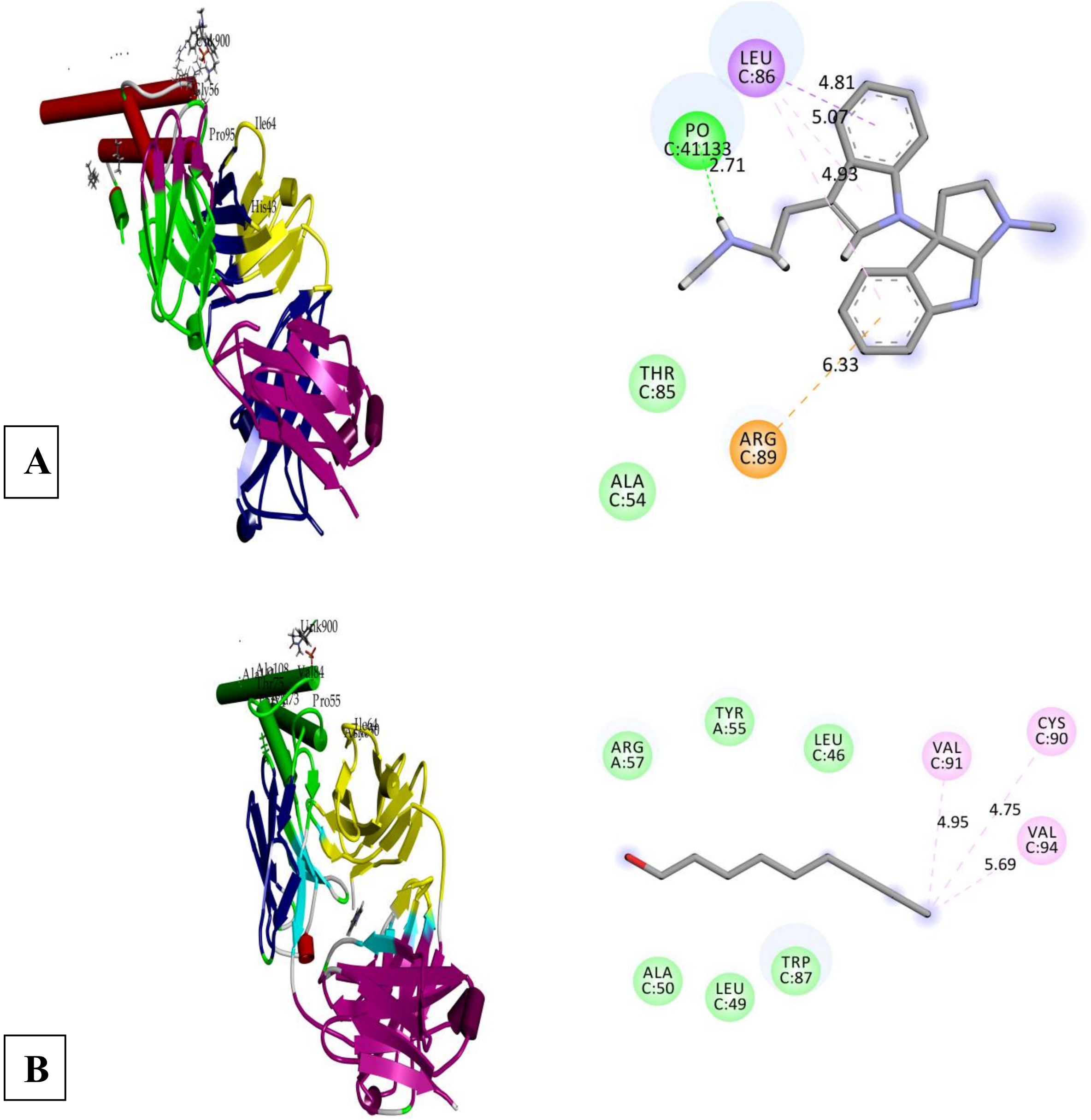

Figure S1: 3D and 2D interactions of psychotriasine (A) and diazepam (B) with the potassium channel receptor (PDB: 4UUJ) for anxiolytic activity.

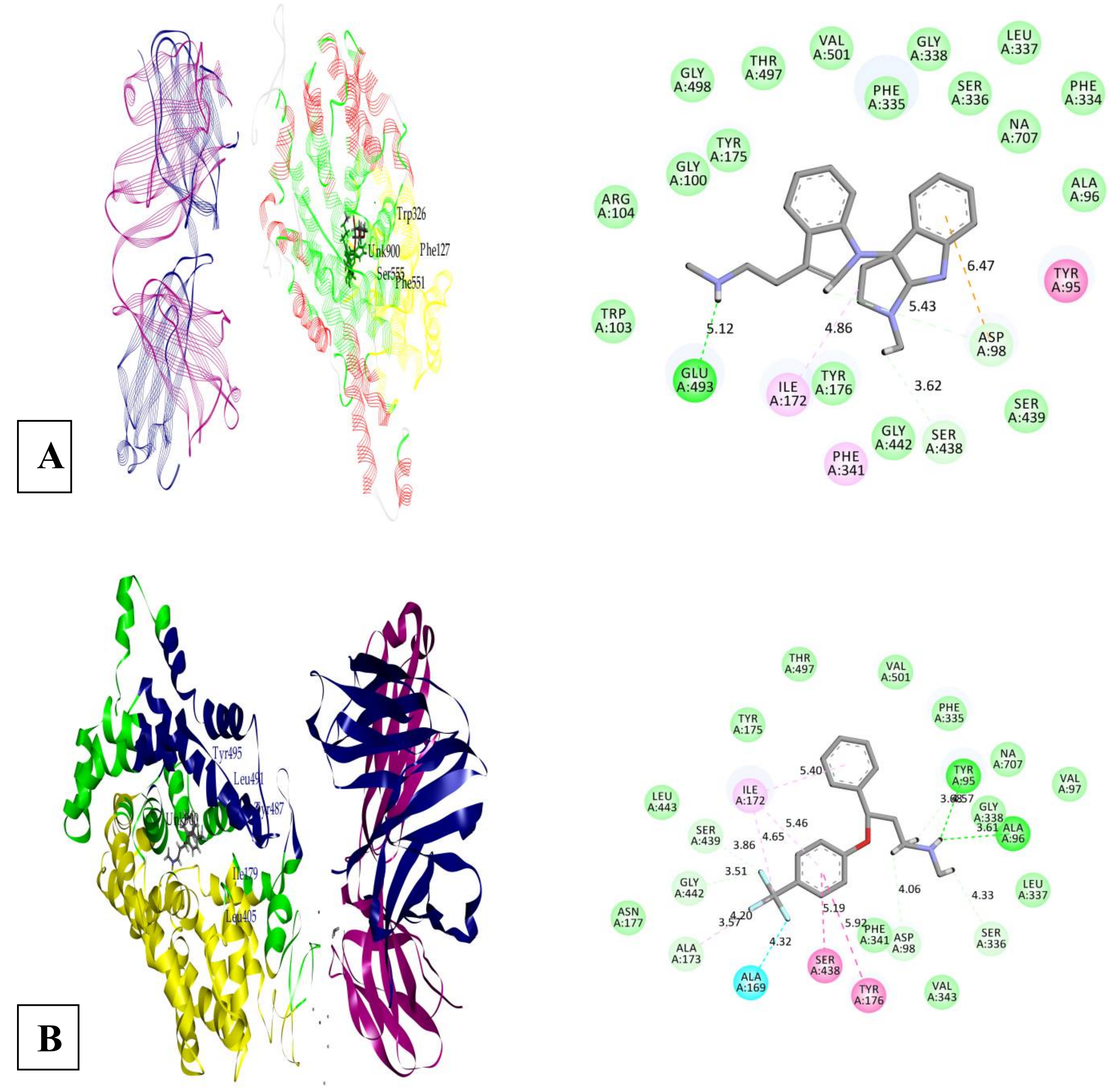

Figure S2: 3D and 2D interactions of psychotriasine (A) and fluoxetine (B) with the human serotonin receptor (PDB: 5I6X) for antidepressant activity.

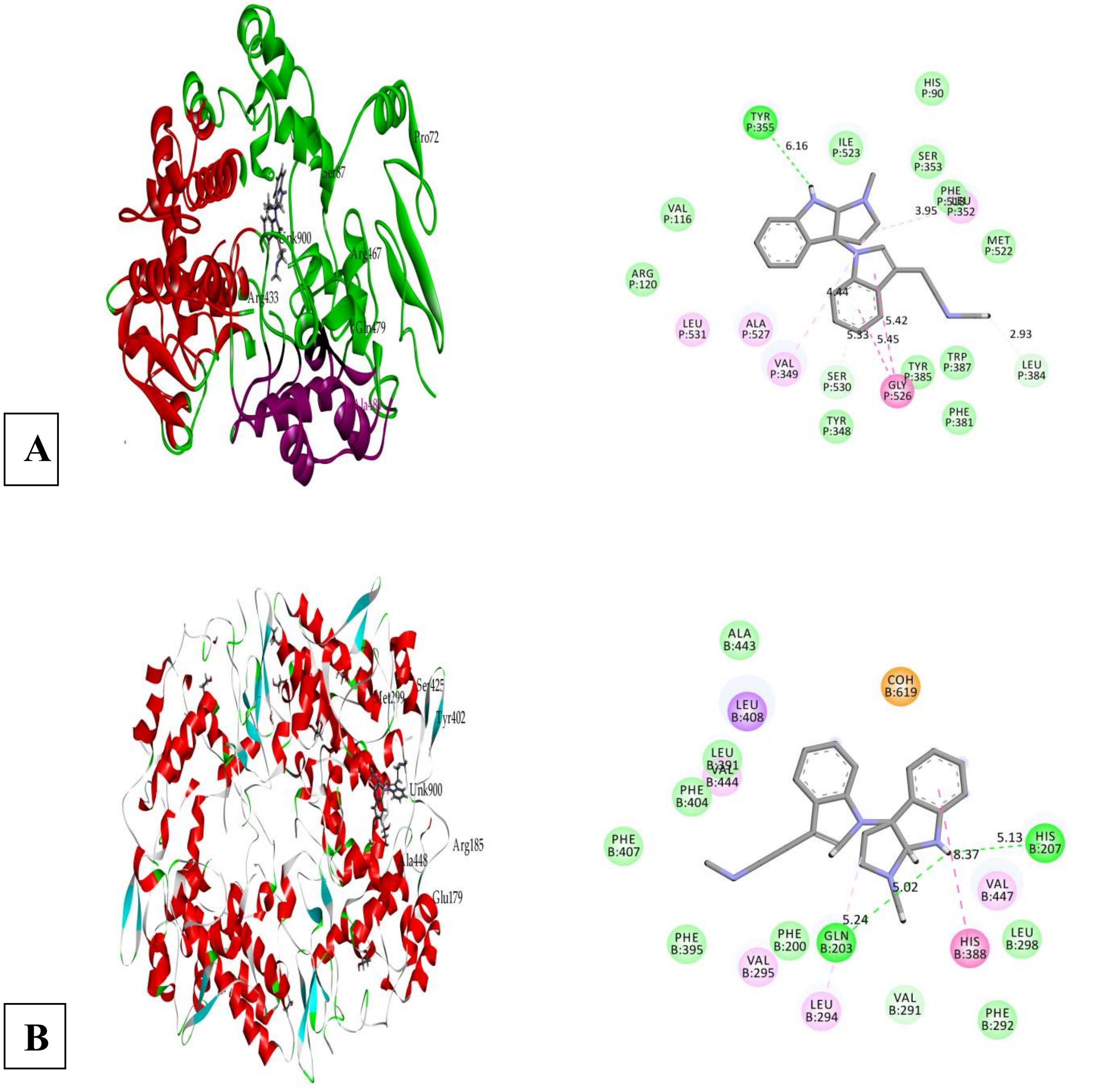

Figure S3: 3D and 2D interactions of psychotriasine with COX-1 (PDB: 2OYE, A) and COX-2 (PDB: 3HS5, B) enzyme for anti-nociceptive activity.

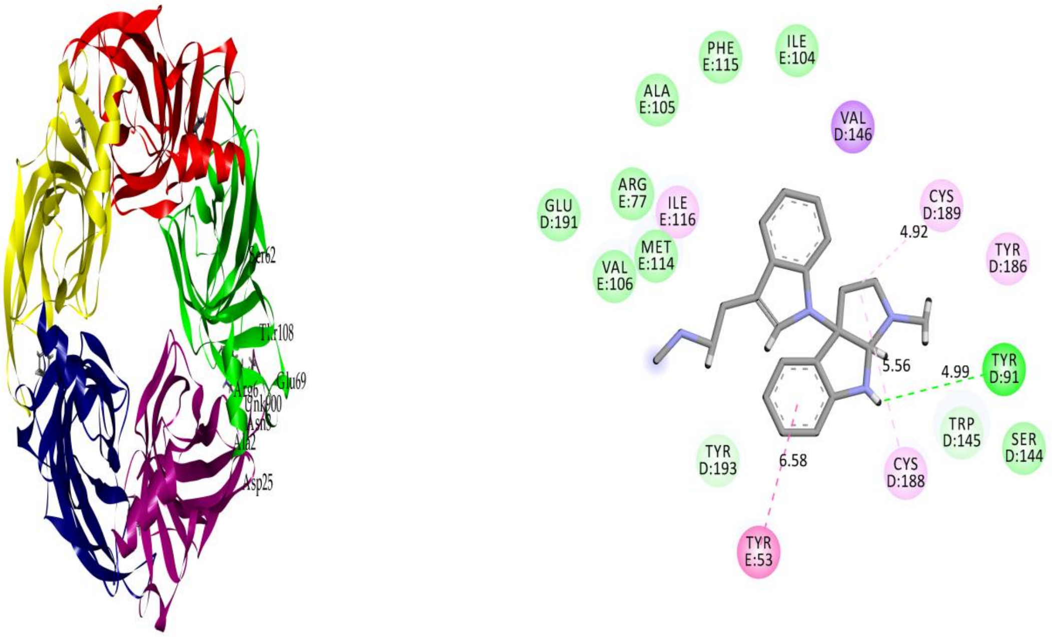

Figure S4: 3D and 2D interactions of psychotriasine with 5-HT3 receptor (PDB: 5AIN) for antidiarrheal activity.

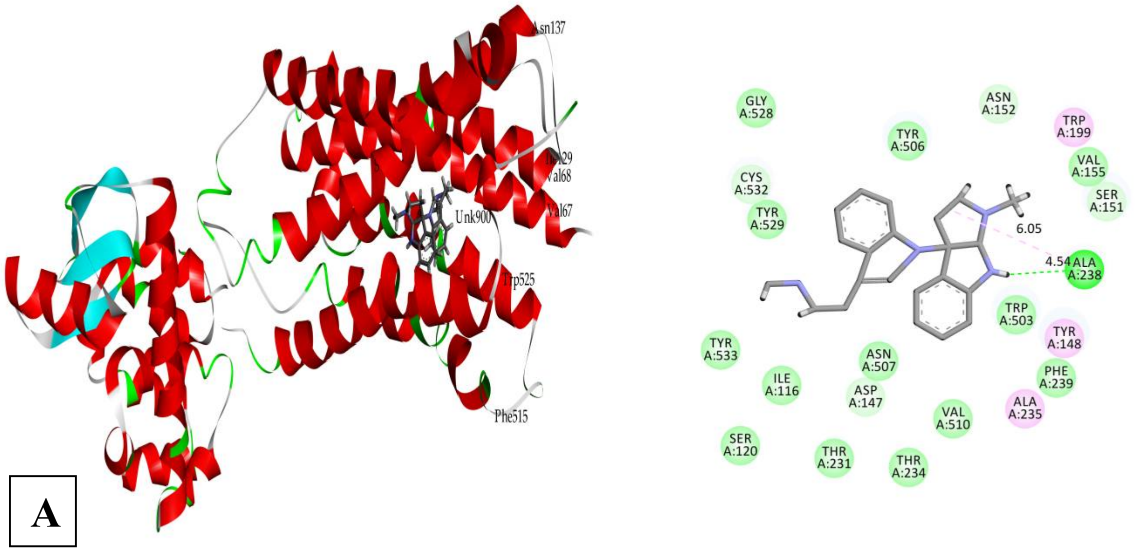

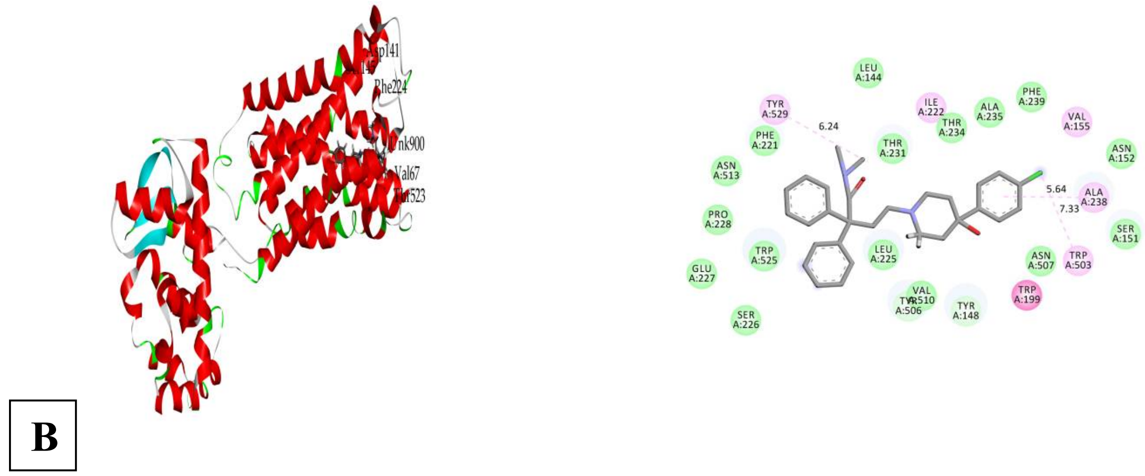

Figure S5: 3D and 2D interactions of psychotriasine (A) and loperamide (B) with M3 muscarinic acetylcholine receptor (PDB: 4U14) for anti-diarrheal activity.

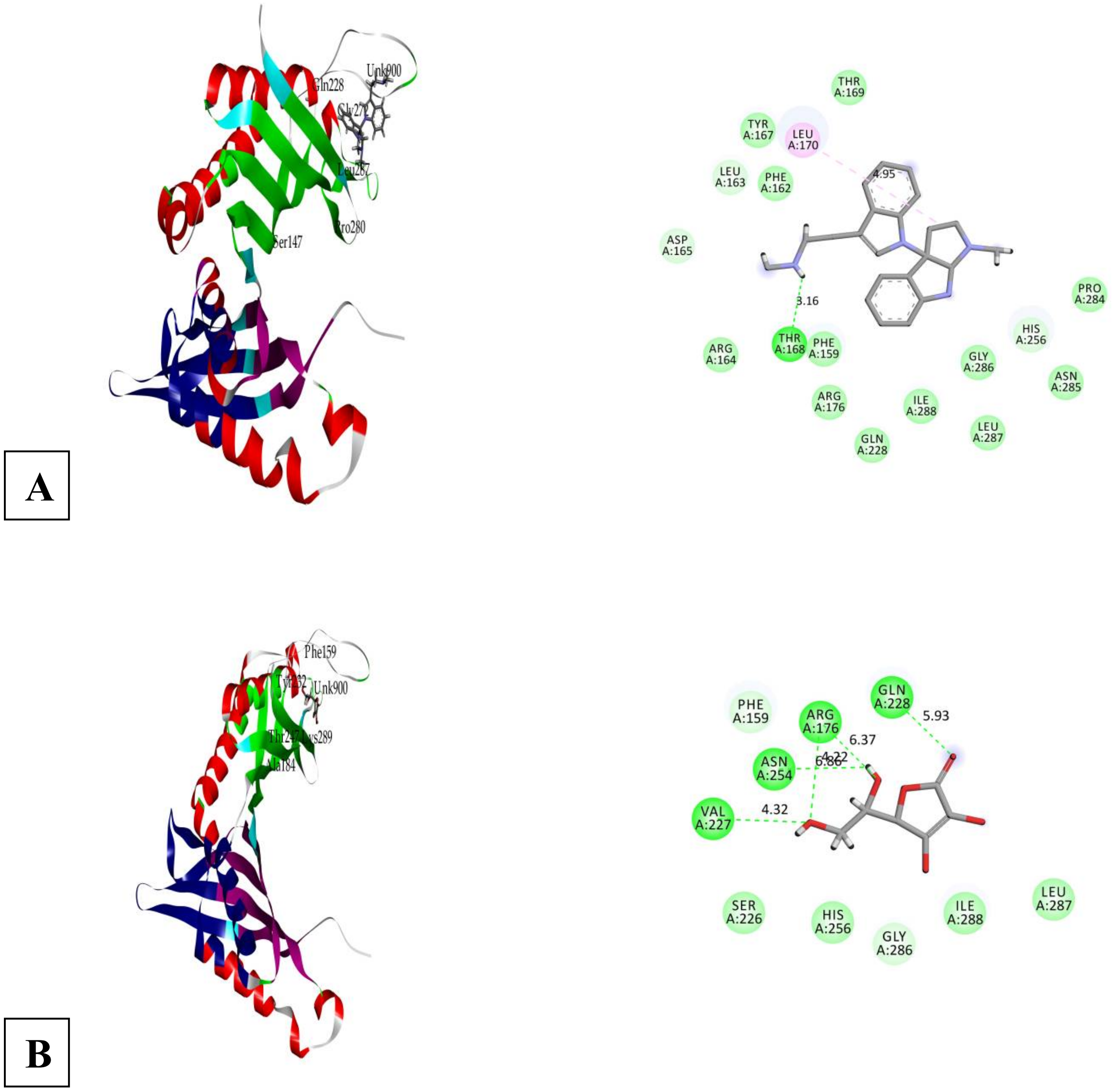

Figure S6: 3D and 2D interactions of psychotriasine (A) and Ascorbic acid (B) with the urate oxidase (PDB: 1R4U) for antioxidant activity.

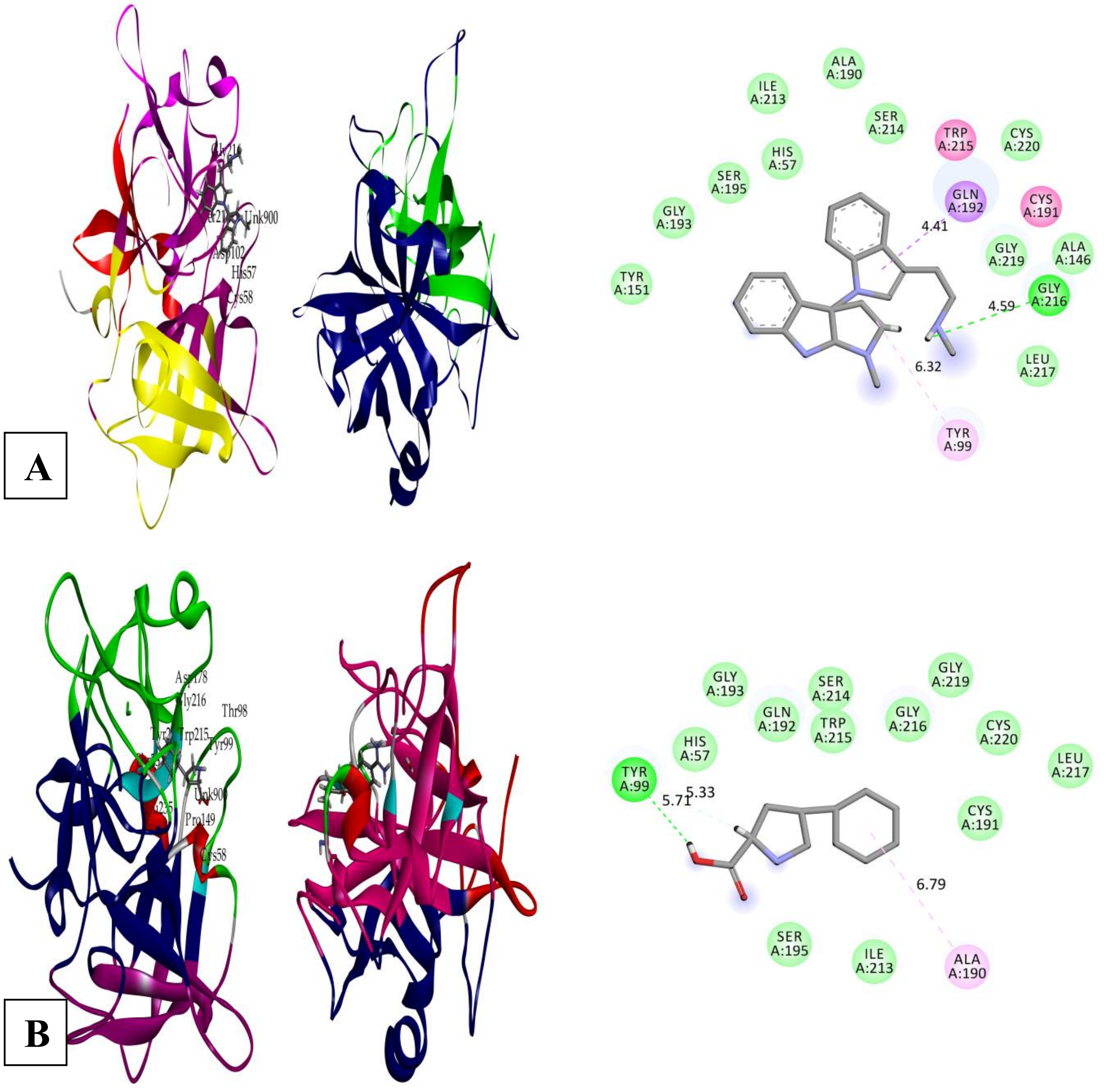

Figure S7: 3D and 2D interactions of psychotriasine (A) and streptokinase (B) with the human tissue plasminogen activator (PDB: 1A5H) for thrombolytic activity.

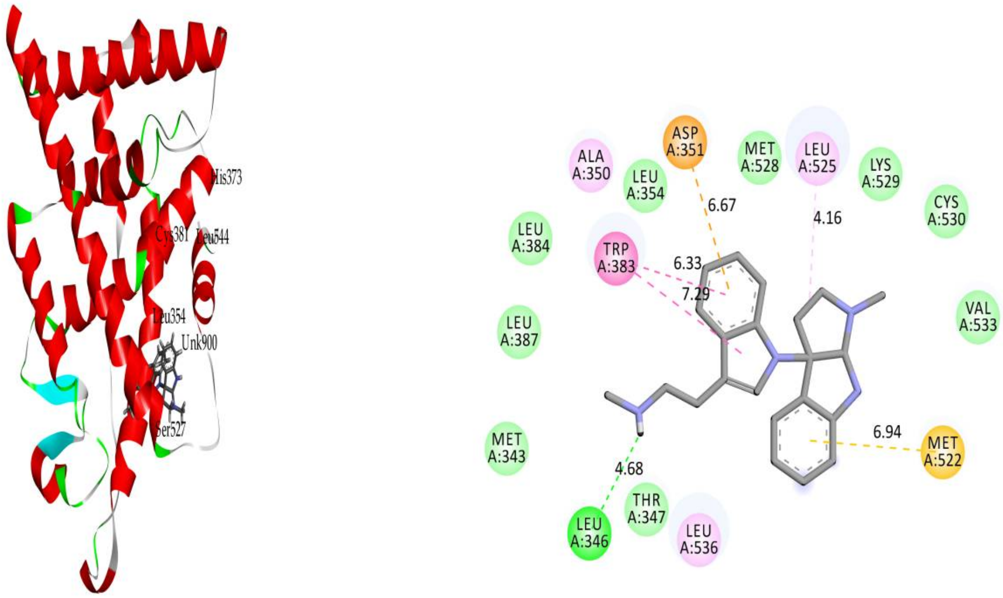

Figure S8: 3D and 2D interactions of psychotriasine with the human estrogen receptor (PDB: 3ERT) for cytotoxic activity.

References

1. Auwal, M. S.; Saka, S.; Mairiga, I. A.; Sanda, K. A.; Shuaibu, A.; Ibrahim, A., Preliminary phytochemical and elemental analysis of aqueous and fractionated pod extracts of *Acacia nilotica* (Thorn mimosa). *Vet Res Forum* **2014,** 5, (2), 95-100.
2. Evans, W. C., *Trease and evans' pharmacognosy E-book*. Elsevier Health Sciences: 2009.
3. Hossain, M. S.; Reza, A.; Rahaman, M. M.; Nasrin, M. S.; Rahat, M. R. U.; Islam, M. R.; Uddin, M. J.; Rahman, M. A., Evaluation of morning glory (*Jacquemontia tamnifolia* (L.) Griseb) leaves for antioxidant, antinociceptive, anticoagulant and cytotoxic activities. *J Basic Clin Physiol Pharmacol* **2018,** 29, (3), 291-299.
